# An Asymmetric Contrastive Loss for Handling Imbalanced Datasets

**DOI:** 10.3390/e24091303

**Published:** 2022-09-15

**Authors:** Valentino Vito, Lim Yohanes Stefanus

**Affiliations:** Faculty of Computer Science, Universitas Indonesia, Depok 16424, Indonesia

**Keywords:** asymmetric loss, class imbalance, contrastive loss, entropy, focal loss

## Abstract

Contrastive learning is a representation learning method performed by contrasting a sample to other similar samples so that they are brought closely together, forming clusters in the feature space. The learning process is typically conducted using a two-stage training architecture, and it utilizes the contrastive loss (CL) for its feature learning. Contrastive learning has been shown to be quite successful in handling imbalanced datasets, in which some classes are overrepresented while some others are underrepresented. However, previous studies have not specifically modified CL for imbalanced datasets. In this work, we introduce an asymmetric version of CL, referred to as ACL, in order to directly address the problem of class imbalance. In addition, we propose the asymmetric focal contrastive loss (AFCL) as a further generalization of both ACL and focal contrastive loss (FCL). The results on the imbalanced FMNIST and ISIC 2018 datasets show that the AFCL is capable of outperforming the CL and FCL in terms of both weighted and unweighted classification accuracies.

## 1. Introduction

Class imbalance is a major obstacle occurring within a dataset when certain classes in the dataset are overrepresented (referred to as majority classes), while some are underrepresented (referred to as minority classes). This can be problematic for a large number of classification models. A deep learning model such as a convolutional neural network (CNN) might not be able to properly learn from the minority classes. Consequently, the model would be less likely to correctly identify minority samples as they occur. This is especially crucial in medical imaging, since a model that cannot identify rare diseases would not be effective for diagnostic purposes. For example, the ISIC 2018 dataset [1,2] is an imbalanced medical dataset that consists of images of skin lesions that appear in various frequencies during screening.

To produce a less imbalanced dataset, it is possible to resample the dataset by either increasing the number of minority samples [3,4,5,6] or decreasing the number of majority samples [7,8,9,10]. Other methods for handling class imbalance include substituting the standard cross-entropy (CE) loss for a more suitable loss, such as the focal loss (FL). Lin et al. [11] modified the CE loss into FL so that minority classes can be prioritized. This is done by ensuring that the model focuses on samples that are harder to classify during model training. Recent studies also unveiled the potential of contrastive learning as a way to combat imbalanced datasets [12,13,14,15].

Contrastive learning is performed by contrasting a sample (called an *anchor*) to other similar samples (called positive samples) so that they are mapped closely together in the feature space. As a consequence, dissimilar samples (called negative samples) are pushed away from the anchor, forming clusters in the feature space based on similarity. In this research, contrastive learning is done using a two-stage training architecture, which utilizes the contrastive loss (CL) formulated by Khosla et al. [16]. This formulation of CL is supervised, and it can contrast the anchor to multiple positive samples belonging to the same class. This is unlike self-supervised contrastive learning [17,18,19,20], which contrasts the anchor to only one positive sample in the mini-batch.

In this work, we propose a modification of supervised CL that is referred to as the asymmetric contrastive loss (ACL). Unlike CL, the ACL is able to directly contrast the anchor to its negative samples so that they are pushed apart in the feature space. This becomes important when a rare sample has no other positive samples in the mini-batch. To our knowledge, we are the first to modify the supervised version of CL in order to address class imbalance, effectively augmenting several studies performed previously in [12,13]. The proposed ACL is aimed toward improving the effectiveness of the two-stage architecture originally presented in [12,13], especially in the feature learning aspect. In addition, the ACL is designed as a generalization of CL, and thus, it provides more flexibility and tuning opportunities as a loss function.

We also consider the asymmetric variant of the focal contrastive loss (FCL) [21], which is called the asymmetric focal contrastive loss (AFCL). Using FMNIST and ISIC 2018 as datasets, experiments were performed to test the performance of both the ACL and AFCL in binary classification tasks. It was observed that the AFCL was superior to the CL and FCL in multiple class imbalance scenarios, provided that suitable hyperparameters were used. In addition, this work provides a streamlined survey of the literature related to entropy and loss functions.

## 2. Related Work

Several studies have been conducted in recent years on the application of contrastive losses to imbalanced datasets. On Siamese networks, for example, Wang et al. [14] and Alenezi et al. [15] proposed the novel focal CL and W-shaped CL, respectively. Their methods managed to achieve state-of-the-art performance in handling the class imbalance problem, wherein Wang et al. used satellite images and Alenezi et al. used skin lesion images as datasets. Their CL functions had a different form from that of the supervised CL of Khosla et al. [16], which is the CL that upon which our study is based.

Marrakchi et al. [12] and Chen et al. [13] independently adopted supervised CL to combat class imbalance in the medical domain. They both used a two-stage architecture consisting of (1) feature learning using CL, followed by (2) fine-tuning using classification loss. Their architectures were almost identical; they differed only in the type of loss function during fine-tuning (Marrakchi et al. used cross-entropy loss, while Chen et al. used focal loss). One limitation present in these studies was that CL was not modified further to deal with imbalance and was implemented as is. Therefore, our aim is to generalize CL in order to effectively learn from imbalanced datasets using the aforementioned two-stage architecture.

In this paper, we present a novel CL referred to as the ACL, and we include its focal-based variant, AFCL. Our motivation for introducing the losses comes from both the asymmetric loss due to Ben-Baruch et al. [22] and the focal contrastive loss due to Zhang et al. [21], whose explanations are provided in Section 3. Although these losses were proposed for different applications (fine-tuning and multi-label classification, respectively), it turns out that these ideas can be applied to our goal of modifying CL so as to handle imbalance.

## 3. Background on Entropy and Loss Functions

In this section, we provide a literature review on the basics of information theory and loss functions for easy reference.

### 3.1. Entropy, Information, and Divergence

Introduced by Shannon [23], entropy provides a measure of the amount of information contained in a random variable, usually in bits. The *entropy* H(X) of a random variable *X* is given by the formula

(1) $$H\left(X\right)=E_{P_{X}}\left[-\log(P_{X}\left(X\right))\right].$$

Given two random variables *X* and *Y*, their *joint entropy* H(X,Y) is the entropy of the joint random variable (X,Y):

(2) $$H\left(X,Y\right)=E_{P_{\left(X,Y\right)}}\left[-\log(P_{\left(X,Y\right)}\left(X,Y\right))\right].$$

In addition, the *conditional entropy* H(Y∣X) is defined as

(3) $$H\left(Y|X\right)=E_{P_{\left(Y,X\right)}}\left[-\log(P_{Y|X}\left(Y|X\right)\right].$$

Conditional entropy is used to measure the average amount of information contained in *Y* when the value of *X* is given. Conditional entropy is bounded above by the original entropy; that is, H(Y∣X)≤H(Y), with equality if and only if *X* and *Y* are independent [24]. The formulas for entropy, joint entropy, and conditional entropy can be derived via an axiomatic approach [25,26].

The *mutual information* I(X;Y) is a measure of dependence between random variables *X* and *Y* [27]. It provides the amount of information about one random variable provided by the other random variable, and it is defined by

(4) I(X;Y)=H(X)−H(X∣Y)=H(Y)−H(Y∣X).

Mutual information is symmetric. In other words, I(X;Y)=I(Y;X). Mutual information is also nonnegative (I(X;Y)≥0), and I(X;Y)=0 if and only if *X* and *Y* are independent [24].

The dissimilarity between random variables *X* and $X^{\prime }$ on the same space X can be measured using the notion of *KL-divergence*:

(5) $$D_{\mathrm{KL}}\left(X\;∥\;X^{\prime }\right)=E_{P_{X}}\left[\log\left(\frac{P_{X}\left(X\right)}{P_{X^{\prime }}\left(X\right)}\right)\right].$$

Similarly to mutual information, KL-divergence is nonnegative ($D_{\mathrm{KL}}\left(X\;∥\;X^{\prime }\right)\geq 0$), and $D_{\mathrm{KL}}\left(X\;∥\;X^{\prime }\right)=0$ if and only if $X=X^{\prime }$ [24]. Unlike mutual information, KL-divergence is asymmetric, so $D_{\mathrm{KL}}\left(X\;∥\;X^{\prime }\right)$ and $D_{\mathrm{KL}}\left(X^{\prime }\;∥\;X\right)$ are not necessarily equal.

### 3.2. Cross-Entropy and Focal Loss

Given random variables *X* and $\hat{X}$ on the same space X, their *cross-entropy* $H(X;\hat{X})$ is defined as [28]:

(6) $$H\left(X;\hat{X}\right)=E_{P_{X}}\left[-\log(P_{\hat{X}}\left(X\right)\right].$$

Cross-entropy is the average number of bits needed to encode the true distribution *X* when its estimate $\hat{X}$ is provided [29]. A small value of $H(X;\hat{X})$ implies that $\hat{X}$ is a good estimate for *X*. Cross-entropy is connected to KL-divergence via the following identity:

(7) $$H\left(X;\hat{X}\right)=H\left(X\right)+D_{\mathrm{KL}}\left(X\;∥\;\hat{X}\right).$$

When $\hat{X}=X$, the equality $H\left(X;\hat{X}\right)=H\left(X\right)$ holds.

Now, the cross-entropy loss and focal loss are provided within the context of a binary classification task consisting of two classes labeled 0 and 1. Suppose that y∈{0,1} denotes the ground-truth class and p∈[0,1] denotes the estimated probability for the class labeled 1. The value of 1−p is then the estimated probability for the class labeled 0. The *cross-entropy (CE) loss* is given by

$$\begin{array}{cc} L_{\mathrm{CE}} & =-y\log(p)-(1-y)\log(1-p)\\ \; & =\left\{\begin{array}{cc} -\log(p) & y=1,\\ -\log(1-p) & y=0. \end{array}\right. \end{array}$$

If y=1, then the loss $L_{\mathrm{CE}}$ is zero when p=1. On the other hand, if y=0, then the loss is zero when 1−p=1. In either case, the CE loss is minimized when the estimated probability of the true class is maximized, which is the desired property of a good classification model.

The *focal loss* (FL) [11] is a modification of the CE loss introduced to put more focus on hard-to-classify examples. It is given by the following formula:

(8) $$L_{\mathrm{foc}}=-y{\left(1-p\right)}^{\gamma }\log\left(p\right)-\left(1-y\right)p^{\gamma }\log\left(1-p\right).$$

**Figure 1:**
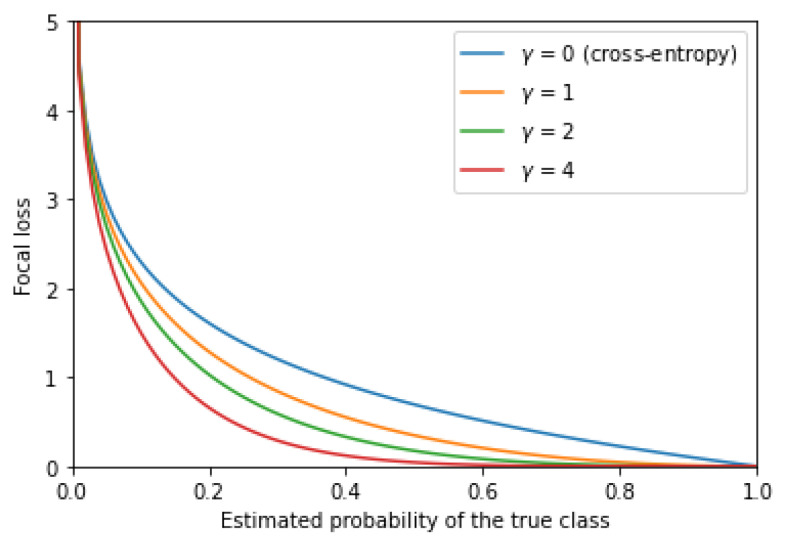
A graph illustrating the focal loss given the predicted probability of the ground-truth class, with varying values of γ.

The parameter γ in $L_{\mathrm{foc}}$ is known as the *focusing parameter*. Choosing a larger value of γ would push the model to focus on training from the misclassified examples. For instance, suppose that γ=4 and denote the estimated probability of the true class by $p_{t}$. The graph in Figure 1 shows that when $p_{t}>0.5$, the FL is quite small. Hence, the model would be less concerned about learning from an example when $p_{t}$ is already sufficiently large. FL is a useful choice when class imbalance exists, as it can help the model focus on the less represented samples within the dataset.

### 3.3. Asymmetric Loss

For multi-label classification with *K* labels, let $y_{i}\in \left\{0,1\right\}$ be the ground truth for class *i* and let $p_{i}\in \left[0,1\right]$ be its estimated probability obtained by the model. The aggregate classification loss is then

(9) $$L=\sum_{i=1}^{K}L_{i},$$

where

(10) $$L_{i}=-y_{i}L_{i}^{+}-\left(1-y_{i}\right)L_{i}^{-}.$$

If FL is the chosen type of loss, $L_{i}^{+}$ and $L_{i}^{-}$ are set as follows:

(11) $$L_{i}^{+}={\left(1-p_{i}\right)}^{\gamma }\log\left(p_{i}\right)\;\mathrm{and}\;L_{i}^{-}=p_{i}^{\gamma }\log\left(1-p_{i}\right).$$

In a typical multi-label dataset, the ground truth $y_{i}$ has value 0 for the majority of classes *i*. Consequently, the negative terms $L_{i}^{-}$ dominate in the calculation of the aggregate loss L. *Asymmetric loss* (ASL) [22] is a proposed solution to this problem. ASL emphasizes the contribution of the positive terms by modifying the losses of (11) to

(12) $$L_{i}^{+}={\left(1-p_{i}\right)}^{\gamma^{+}}\log\left(p_{i}\right)$$

and

(13) $$L_{i}^{-}={\left(p_{i}^{\left(m\right)}\right)}^{\gamma^{-}}\log\left(1-p_{i}^{\left(m\right)}\right),$$

where $\gamma^{+},\gamma^{-}$ are hyperparameters and $p_{i}^{\left(m\right)}$ is the *shifted probability* of $p_{i}$ obtained from the *probability margin* m≥0 via the formula

(14) $$p_{i}^{\left(m\right)}=\max\left(p_{i}-m,0\right).$$

This shift helps decrease the contribution of $L_{i}^{-}$. Indeed, if we set m=1, then $L_{i}^{-}=0$.

### 3.4. Contrastive Loss

Contrastive learning is a learning method for learning representations from data. A supervised approach of contrastive learning was introduced by Khosla et al. [16] to learn from a set of sample–label pairs ${\left\{\left(x_{i},y_{i}\right)\right\}}_{i=1}^{N}$ in a mini-batch of size *N*. The samples $x_{i}$ are fed through a feature encoder Enc(·) and a projection head Proj(·) in succession to obtain features $z_{i}=\mathrm{Proj}\left(\mathrm{Enc}\left(x_{i}\right)\right)$. The feature encoder extracts features from $x_{i}$, whereas the projection head projects the features into a lower dimension and applies $\ell_{2}$-normalization so that $z_{i}$ lies in the unit hypersphere. In other words, ${∥z_{i}∥}_{2}=1$.

A pair $\left(z_{i},z_{j}\right)$, where i≠j, is referred to as a *positive pair* if the features share the same class label ($y_{i}=y_{j}$), and it is a *negative pair* if the features have different class labels ($y_{i}\neq y_{j}$). Contrastive learning aims to maximize the similarity between $z_{i}$ and $z_{j}$ whenever they form a positive pair and minimize their similarity whenever they form a negative pair. This similarity is measured with cosine similarity [29]:

(15) $$\kappa \left(z_{i},z_{j}\right)=\frac{z_{i}\cdot z_{j}}{{∥z_{i}∥}_{2}{∥z_{j}∥}_{2}}=z_{i}\cdot z_{j}.$$

From the above equation, we have $\kappa \left(z_{i},z_{j}\right)\in \left[-1,1\right]$. In addition, $\kappa (z_{i},z_{j})=1$ when $z_{i}=z_{j}$, and $\kappa (z_{i},z_{j})=-1$ when $z_{i}$ and $z_{j}$ form a $180^{\circ }$ angle.

Fixing $z_{i}$ as the anchor, let $A_{i}=\left\{z_{k}|k\neq i\right\}$ be the set of features other than $z_{i}$ and let $P_{i}=\left\{z_{k}\in A_{i}|y_{k}=y_{i}\right\}$ be the set of $z_{k}$ such that $\left(z_{i},z_{k}\right)$ is a positive pair. The predicted probability $p_{ij}$ that $z_{i}$ and $z_{j}$ belong to the same class is obtained by applying the softmax function to the the set of similarities between $z_{i}$ and $z_{k}\in A_{i}$:

(16) $$p_{ij}=\frac{\exp(z_{i}\cdot z_{j}/\tau )}{\sum_{z_{k}\in A_{i}}\exp\left(z_{i}\cdot z_{k}/\tau \right)},$$

where τ is referred to as the *temperature parameter*. Since our goal is to maximize $p_{ij}$ whenever $z_{j}\in P_{i}$, the *contrastive loss* that is to be minimized is formulated as

(17) $$L_{\mathrm{con}}=-\sum_{i=1}^{n}\frac{1}{|P_{i}|}\sum_{z_{j}\in P_{i}}\log\left(p_{ij}\right).$$

Information-theoretical properties of $L_{\mathrm{con}}$ are given in [21], for which we provide a summary. Let *X*, *Y*, and *Z* denote random variables of the samples, labels, and features, respectively. The following theorem states that $L_{\mathrm{con}}$ is positively proportional to H(Z∣Y)−H(Z) under the assumption that no class imbalance exists.

**Theorem** **1**  (Zhang et al. [21])**.** *Assuming that features are $\ell_{2}$-normalized and the dataset is balanced,*

(18) $$L_{\mathrm{con}}\propto H\left(Z|Y\right)-H\left(Z\right).$$

Theorem 1 implies that minimizing $L_{\mathrm{con}}$ is equivalent to minimizing the conditional entropy H(Z∣Y) and maximizing the feature entropy H(Z). Since I(Z;Y)=H(Z)−H(Z∣Y), minimizing $L_{\mathrm{con}}$ is equivalent to maximizing the mutual information I(Z;Y) between features *Z* and class labels *Y*. In other words, contrastive learning aims to extract the maximum amount of information from class labels and encode it in the form of features.

After the features are extracted, a classifier Clas(·) is assigned to convert $z_{i}$ into a prediction ${\hat{y}}_{i}=\mathrm{Clas}\left(z_{i}\right)$ of the class label. The random variable of predicted class labels is denoted by $\hat{Y}$.

For the next theorem, the definition of *conditional cross-entropy* $H(Y;\hat{Y}|Z)$ is given as follows:

(19) $$H\left(Y;\hat{Y}|Z\right)=E_{P_{\left(Y,Z\right)}}\left[-\log(P_{\left(\hat{Y},Z\right)}\left(Y,Z\right)\right].$$

Conditional CE measures the average amount of information needed to encode the true distribution *Y* using its estimate $\hat{Y}$ given the value of *Z*. A small value of $H(Y;\hat{Y}|Z)$ implies that $\hat{Y}$ is a good estimate for *Y* given *Z*.

**Theorem** **2**  (Zhang et al. [21])**.** *Assuming that features are $\ell_{2}$-normalized and the dataset is balanced,*

(20) $$L_{\mathrm{con}}\propto \inf\;H\left(Y;\hat{Y}|Z\right)-H\left(Y\right),$$

*where the infimum is taken over classifiers.*

Theorem 2 implies that minimizing $L_{\mathrm{con}}$ will minimize the infimum of conditional cross-entropy $H(Y;\hat{Y}|Z)$ taken over classifiers. As a consequence, contrastive learning is able to encode features in *Z* such that the best classifier can produce a good estimate of *Y* given the information provided by the feature encoder.

The formula for $L_{\mathrm{con}}$ can be modified so as to resemble the focal loss, resulting in a loss function known as the *focal contrastive loss* (FCL) [21]:

(21) $$L_{\mathrm{FC}}=-\sum_{i=1}^{n}\frac{1}{|P_{i}|}\sum_{z_{j}\in P_{i}}\left(1-p_{ij}\right)\log\left(p_{ij}\right).$$

## 4. Proposed Loss Functions and Architecture

In this section, our proposed modification of the contrastive loss, which is called the asymmetric contrastive loss, is introduced. In addition, the architecture of the model in which the contrastive losses are implemented is explained. Our proposed asymmetric loss function is novel, while the architecture is obtained from [12,13] with no changes made. Thus, our contribution lies simply in the change of the loss function.

### 4.1. Asymmetric Contrastive Loss

In (17), the inside summation of the contrastive loss is evaluated over $P_{i}$. Consequently, according to (16), each anchor $z_{i}$ is contrasted with vectors $z_{j}$ that belong to the same class. This does not present a problem when the mini-batch contains plenty of examples from each class. However, the calculated loss may not give each class a fair contribution when some classes are less represented in the mini-batch.

**Figure 2:**
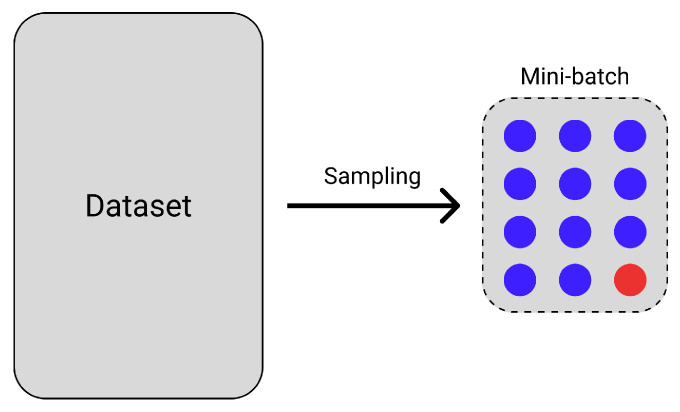
A mini-batch consisting of 11 examples with a blue-colored class label and one example with a red-colored class label.

In Figure 2, a sampled mini-batch consists of 11 examples with a blue-colored class label and one example with a red-colored class label. When the anchor $z_{i}$ is the representation of the red-colored sample, $z_{i}$ does not directly contribute to the calculation of $L_{\mathrm{con}}$, since $P_{i}$ is empty. In other words, $z_{i}$ cannot be contrasted to any other sample in the mini-batch. This scenario is likely to happen when the dataset is imbalanced, and it motivates us to modify CL so that each anchor $z_{i}$ can also be contrasted with $z_{j}$ not belonging to the same class.

Let $N_{i}=A_{i}\backslash P_{i}$ be the set of vectors $z_{k}$ such that $\left(z_{i},z_{k}\right)$ is a negative pair. Motivated by the $L_{i}^{+}$ and $L_{i}^{-}$ of (10), we define

(22) $$L_{i}^{+}=\frac{1}{|P_{i}|}\sum_{z_{j}\in P_{i}}\log\left(p_{ij}\right)$$

and

(23) $$L_{i}^{-}=\frac{1}{|N_{i}|}\sum_{z_{j}\in N_{i}}\log\left(1-p_{ij}\right),$$

where $p_{ij}=\exp\left(z_{i}\cdot z_{j}/\tau \right)/\sum_{z_{k}\in A_{i}}\exp\left(z_{i}\cdot z_{k}/\tau \right)$. The loss function $L_{i}^{+}$ contrasts $z_{i}$ to vectors in $P_{i}$, whereas $L_{i}^{-}$ contrasts $z_{i}$ to vectors in $N_{i}$. The resulting *asymmetric contrastive loss* (ACL) is given by the formula

(24) $$L_{\mathrm{AC}}=-\sum_{i=1}^{n}\left(L_{i}^{+}+\eta L_{i}^{-}\right),$$

where η≥0 is a fixed hyperparameter. If η=0, then $L_{\mathrm{AC}}=L_{\mathrm{con}}$. Hence, ACL is a generalization of CL.

When the batch size is set to a large number (over 100, for example), the value $p_{ij}$ tends to be very small. This causes $L_{i}^{-}$ to be much smaller than $L_{i}^{+}$. In order to balance their contribution to the total loss $L_{\mathrm{AC}}$, a large value for η is usually chosen (between 60 and 300 in our experiment).

In summary, we propose ACL in order to (1) generalize the CL via the addition of a summation over negative samples and (2) specifically address the problem of class imbalance. ACL is intended to be both more flexible and robust to imbalances than the vanilla CL.

### 4.2. Asymmetric Focal Contrastive Loss

Following the formulation of $L_{\mathrm{FC}}$ in (21), $L_{i}^{+}$ can be modified to have the following formula:

(25) $$L_{i}^{+}=\frac{1}{|P_{i}|}\sum_{z_{j}\in P_{i}}{\left(1-p_{ij}\right)}^{\gamma }\log\left(p_{ij}\right).$$

Using this loss, the *asymmetric focal contrastive loss* (AFCL) is then given by

(26) $$L_{\mathrm{AFC}}=-\sum_{i=1}^{n}\left(L_{i}^{+}+\eta L_{i}^{-}\right),$$

where $L_{i}^{-}=\frac{1}{|N_{i}|}\sum_{z_{j}\in N_{i}}\log\left(1-p_{ij}\right)$. We do not modify $L_{i}^{-}$ by adding the multiplicative term ${\left(p_{ij}\right)}^{\gamma }$, since $p_{ij}$ is usually too small and would make $L_{i}^{-}$ vanish if the term is added.

We have $L_{\mathrm{AFC}}=L_{\mathrm{FC}}$ when γ=1. Thus, AFCL generalizes the FCL. Unlike with the FCL, we add the hyperparameter γ≥0 to the loss function so as to provide some flexibility to the loss function.

### 4.3. Model Architecture

**Figure 3:**
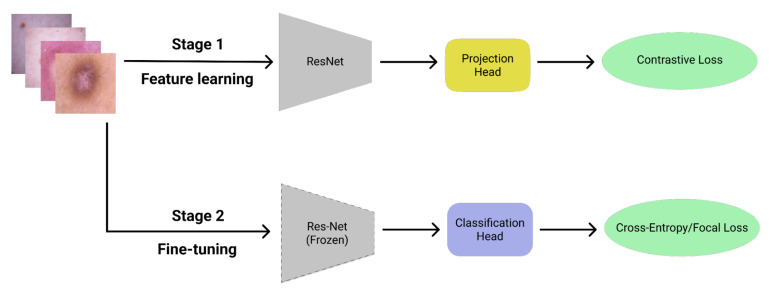
A two-stage training strategy consisting of: (1) feature learning using contrastive loss and (2) classifier fine-tuning using either FL or CE loss.

This section explains the inner workings of the classification model used for the implementation of the contrastive losses. The architecture of the model is taken from [12,13]. The training strategy for the model, as shown in Figure 3, comprises two stages: the feature-learning stage and the fine-tuning stage.

In the first stage, each mini-batch is fed through a feature encoder. We consider either ResNet-18 or ResNet-50 [30] for the architecture of the feature encoder. The output of the feature encoder is projected by the projection head to generate a vector z of length 128. If ResNet-18 is used for the feature encoder, then the projection head consists of two layers of lengths 512 and 128. If ResNet-50 is used, then the two layers are of lengths 2048 and 128. Afterwards, z is $\ell_{2}$-normalized, and the model parameters are updated using some version of the contrastive loss (either CL, FCL, ACL, or AFCL).

After the first stage is complete, the feature encoder is frozen and the projection head is removed. In its place, we have a one-layer classification head that generates the estimated probability that the training sample belongs to a certain class. The parameters of the classification head are updated using either the FL or CE loss. The final classification model is the feature encoder trained during the first stage, together with the classification head trained during the second stage. Since the classification head is a significantly smaller architecture than the feature encoder, the training is mostly focused on the first stage. As a consequence, we typically need a larger number of epochs for the feature-learning stage compared to the fine-tuning stage.

## 5. Experiments

The datasets and settings of our experiments are outlined in this section. We provide and discuss the results of the experiments on the FMNIST and ISIC 2018 datasets. The PyTorch implementation is available on GitHub (https://github.com/valentinovito/Asymmetric-CL, accessed on 8 September 2022).

### 5.1. Datasets

In our experiments, the training strategy outlined in Section 4.3 was applied to two imbalanced datasets. The first was a modified version of the Fashion-MNIST (FMNIST) dataset [31], and the second was the International Skin Imaging Collaboration (ISIC) 2018 medical dataset [1,2].

**Figure 4:**
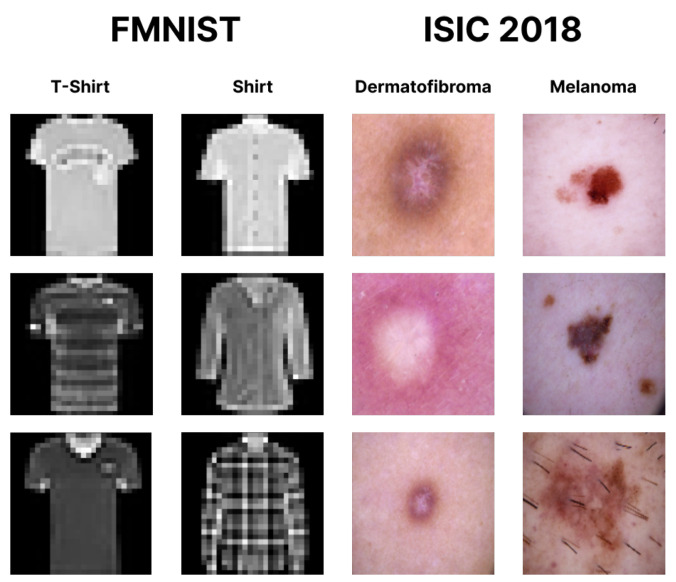
Sample images of the FMNIST and ISIC 2018 datasets.

The FMNIST dataset consisted of low-resolution (28×28 pixels) grayscale images of ten classes of clothing. In this study, we took only two classes to form a binary classification task: the T-shirt and shirt classes. The samples were taken such that the proportion between the T-shirt and shirt images could be imbalanced, depending on the scenario. On the other hand, the ISIC 2018 dataset consisted of high-resolution RGB images of seven classes of skin lesions. As with FMNIST, we used only two classes for the experiments: the melanoma and dermatofibroma classes. Illustrations of the sample images of both datasets are provided in Figure 4.

FMNIST was chosen as our dataset, since, although simple, it is a benchmark dataset for testing deep learning models for computer vision. On the other hand, ISIC 2018 was chosen since it is a domain-appropriate imbalanced dataset for our model. We first applied the model (using AFCL as the loss function) to the more lightweight FMNIST dataset under various class imbalance scenarios. This was conducted to check the appropriate values of the η and γ parameters of the AFCL under different imbalance conditions. Afterwards, the model was applied to the ISIC 2018 dataset using the optimal parameter values obtained during the FMNIST experiments.

### 5.2. Experimental Details

The experiments were conducted using the NVIDIA Tesla P100-PCIE GPU allocated by the Google Colaboratory Pro platform. The models and loss functions were implemented using PyTorch. To process the FMNIST dataset, we used the simpler ResNet-18 architecture as the feature encoder and trained it for 20 epochs. On the other hand, to process the ISIC 2018 dataset, we used the deeper ResNet-50 as the feature encoder and trained it for 40 epochs. For both the FMNIST and ISIC 2018 datasets, the learning rate and batch size were set to $10^{-2}$ and 128, respectively. In addition, the classification head was trained for 10 epochs. The encoder and the classification head were both trained using the Adam optimizer. Finally, the temperature parameter τ of the contrastive loss was set to its default value of 0.07.

The evaluation metrics utilized in the experiment were (weighted) accuracy and unweighted accuracy (UWA), both of which could be calculated from the number of true positives (TP), true negatives (TN), false negatives (FN), and false positives (FP) using the formulas

(27) $$\mathrm{Accuracy}=\frac{\mathrm{TP}+\mathrm{TN}}{\mathrm{TP}+\mathrm{TN}+\mathrm{FN}+\mathrm{FP}}$$

and

(28) $$\mathrm{UWA}=\frac{1}{2}\left(\frac{\mathrm{TP}}{\mathrm{TP}+\mathrm{FN}}+\frac{\mathrm{TN}}{\mathrm{TN}+\mathrm{FP}}\right),$$

respectively. Unlike accuracy, the UWA provided the average of the individual class accuracies regardless of the number of samples in the test set of each class. UWA is an appropriate metric when a dataset is significantly imbalanced [32].

For heavily imbalanced datasets, a high accuracy and low UWA may mean that the model is biased towards classifying samples as part of the majority class. This indicates that the model does not properly learn from the minority samples. In contrast, a lower accuracy with a high UWA indicates that the model takes significant risks to classify some samples as part of the minority class. Our aim was to construct a model that maximized both metrics simultaneously; that is, a model that could learn unbiasedly from both the majority and minority samples with minimal misclassification error.

### 5.3. Experiments Using FMNIST

The data used in the FMNIST experiment comprised 1000 images classified as either a T-shirt or a shirt. The dataset was split 70/30 for model training and testing. The images were augmented using random rotations and random flips. We deployed 11 class imbalance scenarios on the dataset, which controlled the proportion between the T-shirt class and the shirt class. For example, if the proportion was 60:40, then 600 T-shirt images and 400 shirt images were sampled to form the experimental dataset. Our proportions ranged from 50:50 to 98:2.

During the first stage, the ResNet-18 encoder was trained using the AFCL. Afterwards, the classification head was trained using the CE loss during the second stage. As AFCL contains two parameters, η and γ, our goal was to tune each of these parameters independently, keeping the other parameter fixed. First, η was tuned as we set γ=0, followed by the tuning of γ as we set η=0. Each experiment was performed four times in total. The average accuracy and UWA of these four runs are provided in Table 1 (for the tuning of η) and Table 2 (for the tuning of γ).

For the tuning of η, six values of η were experimented on: η∈{0,60,120,180,240,300}. When η=0, the loss function was reduced to the ordinary CL. As observed in Table 1, the optimal value of η tended to be larger when the dataset was moderately imbalanced. As the scenario went from 60:40 to 90:10, the parameter η that maximized accuracy increased in value, from η=0 when the proportion was 60:40 to η=300 when the proportion was 90:10. In general, this indicated that the $L_{i}^{-}$ term of the ACL became more essential to the overall loss as the dataset got more imbalanced, confirming the reasoning contained in Section 4.1.

As seen in Table 2, we experimented on γ∈{0,1,2,4,7,10}, where choosing γ=0 meant that we were using the CL. Although the overall pattern of the optimal γ was less apparent than η of the previous experiment, some insights could still be obtained. When the scenario was between 70:30 and 90:10, the focusing parameter γ was optimally chosen when it was larger than zero. This was in direct contrast to when the proportion was perfectly balanced (50:50), where γ=0 was the most optimal parameter. This suggests that a larger value of γ should be considered when class imbalance is significantly present within a dataset.

When the dataset was balanced, however, our experiments suggested that neither asymmetry nor focality was markedly helpful. Indeed, in the 50:50 scenario, CL already provided the second-best accuracy in Table 1 and the best accuracy in Table 2. In Table 1, the CL was the case where η=0 was chosen. In Table 2, on the other hand, the CL was used when γ=0. Therefore, our proposed loss function works best with imbalanced datasets.

### 5.4. Experiments Using ISIC 2018

From the ISIC 2018 dataset, a total of 1113 melanoma images and 115 dermatofibroma images were combined to create the experimental dataset. As with the previous experiment, the dataset was split 70/30 for training and testing. The images were resized to 128×128 pixels. The ResNet-50 encoder was trained using one of the available contrastive losses, which included the CL/FCL as baselines and the ACL/AFCL as the proposed loss functions. The classification head was trained using FL as the loss function, with its focusing parameter set to γ=2.

The proportion between the melanoma class and the dermatofibroma class in the experimental dataset was close to 90:10. Using the results from Table 1 and Table 2 as a heuristic for determining the optimal parameter values, we set η=300 and γ=2,7. It is worth mentioning that even though γ=2 produced the best accuracy in the FMNIST experiment, the UWA of the resulting model was quite poor. However, we decided to include this value in this experiment for completeness.

The results of this experiment are given in Table 3. As in the previous section, each experiment was conducted four times, so the table lists the average accuracy and UWA of these four runs for each contrastive loss tested. Each run, which included both model training and testing, was completed in roughly 80 min using our computational setup.

From Table 3, CL and ACL performed the worst in terms of UWA and accuracy, respectively. However, ACL gave the best UWA among all losses. This may indicate that the ACL encouraged the model to take the risky approach of classifying some samples as part of the minority class at the expense of accuracy. Overall, AFCL with η=300 and γ=7 emerged as the best loss in this experiment, producing the best accuracy and the second-best UWA behind the ACL. This led us to conclude that the AFCL, with optimal hyperparameters chosen, is superior to the vanilla CL and FCL.

## 6. Conclusions and Future Work

In this work, we introduced an asymmetric version of both contrastive loss (CL) and focal contrastive loss (FCL), which are referred to as ACL and AFCL, respectively. These asymmetric variants of the contrastive loss were proposed to provide more focus on the minority class. The experimental model used was a two-stage architecture consisting of a feature-learning stage and a classifier fine-tuning stage. This model was applied to the imbalanced FMNIST and ISIC 2018 datasets using various contrastive losses. Our results show that the AFCL was able to outperform the CL and FCL in terms of both weighted and unweighted accuracies. On the ISIC 2018 binary classification task, AFCL, with η=300 and γ=7 as hyperparameters, achieved an accuracy of 93.75% and an unweighted accuracy of 74.62%. This is in contrast to the FCL, which achieved 93.07% and 74.34% on both metrics, respectively.

The experiments in this research were conducted using datasets consisting of approximately 1000 images in total. In the future, the experimental model may be applied to larger-scale datasets in order to test its scalability. In addition, other models based on the ACL and AFCL can also be developed for specific datasets, ideally within the realm of multi-class classification.

## Figures and Tables

**Table 1:** The accuracy and UWA (averaged over four independent runs) of 11 class imbalance scenarios using various values of η for the AFCL. The parameter γ was consistently set to 0.

Scenario	Metric	η					
		0	60	120	180	240	300
50:50	Accuracy	78.92	77.83	**79.75**	71.08	77.17	78.83
	UWA	79.00	78.28	**80.32**	72.53	77.87	79.42
55:45	Accuracy	**79.50**	**79.50**	79.33	77.83	77.67	77.75
	UWA	78.70	**79.34**	79.15	77.17	78.21	76.50
60:40	Accuracy	**84.50**	82.92	82.42	81.33	82.08	83.17
	UWA	**83.09**	81.82	81.27	79.71	81.74	81.66
65:35	Accuracy	81.50	**83.42**	83.25	81.59	82.58	79.25
	UWA	79.19	**80.91**	80.73	77.92	79.43	75.42
70:30	Accuracy	82.50	84.33	**85.08**	82.08	83.42	83.00
	UWA	78.41	78.26	**80.91**	77.78	79.14	75.11
75:25	Accuracy	86.75	85.17	85.58	85.17	**86.92**	86.58
	UWA	77.87	76.48	77.74	77.03	**78.63**	77.57
80:20	Accuracy	86.00	87.25	87.33	87.92	87.00	**88.25**
	UWA	76.16	74.65	76.94	76.28	**77.49**	76.97
85:15	Accuracy	87.33	87.08	86.75	87.42	87.33	**87.67**
	UWA	**70.08**	66.34	55.77	68.33	69.83	62.83
90:10	Accuracy	90.83	91.00	90.83	90.67	89.50	**91.67**
	UWA	64.91	68.61	66.11	64.02	61.77	**72.58**
95:5	Accuracy	**94.42**	93.33	93.42	94.00	92.83	93.25
	UWA	54.77	**60.70**	54.24	50.00	49.38	54.80
98:2	Accuracy	97.42	97.83	98.08	98.08	**98.33**	98.08
	UWA	52.45	52.66	**55.87**	**55.87**	49.83	52.79

**Table 2:** The accuracy and UWA (averaged over four independent runs) of 11 class imbalance scenarios using various values of γ for the AFCL. The parameter η was consistently set to 0.

Scenario	Metric	γ					
		0	1	2	4	7	10
50:50	Accuracy	**78.08**	74.83	77.08	77.58	76.58	77.50
	UWA	**77.70**	74.84	76.77	77.55	76.55	77.25
55:45	Accuracy	80.17	81.25	80.75	80.00	**81.75**	76.83
	UWA	80.14	81.19	80.69	79.96	**81.70**	76.82
60:40	Accuracy	79.42	78.50	77.92	80.17	**80.67**	80.08
	UWA	**84.42**	83.42	80.00	83.00	82.42	82.92
65:35	Accuracy	**84.42**	83.42	80.00	83.00	82.42	82.92
	UWA	**81.98**	81.22	77.87	80.39	80.68	80.16
70:30	Accuracy	83.75	83.83	82.17	82.58	**84.83**	82.25
	UWA	79.64	79.18	77.82	77.51	**79.67**	78.71
75:25	Accuracy	85.42	**86.17**	84.42	84.83	85.75	86.00
	UWA	76.27	**79.85**	77.08	76.41	77.34	78.47
80:20	Accuracy	89.33	**89.58**	87.67	89.42	87.33	88.00
	UWA	77.59	78.67	78.43	**79.31**	78.97	70.12
85:15	Accuracy	87.42	89.00	88.17	88.33	89.08	**90.08**
	UWA	64.97	72.08	71.99	71.47	71.95	**77.04**
90:10	Accuracy	92.42	92.33	**93.42**	93.25	92.58	91.25
	UWA	64.00	67.94	66.04	74.42	**80.54**	68.35
95:5	Accuracy	94.17	93.17	**95.33**	95.00	94.00	95.09
	UWA	**62.13**	53.11	57.64	59.17	55.22	55.82
98:2	Accuracy	**96.92**	**96.92**	95.00	96.00	**96.92**	96.67
	UWA	**56.59**	51.56	55.61	52.63	53.10	52.98

**Table 3:** The accuracy and UWA (averaged over four independent runs) of the model when trained using various contrastive losses.

Loss Function	Accuracy	UWA
CL [16]	93.00	72.25
FCL [21]	93.07	74.34
ACL (η = 300)	85.94	**75.54**
AFCL (η = 300, γ = 2)	92.39	74.36
AFCL (η = 300, γ = 7)	**93.75**	74.62

## Data Availability

Not applicable.
